# Development and Validation of a Practical Model for Transient Biofilter Performance

**DOI:** 10.3390/biotech11040051

**Published:** 2022-10-29

**Authors:** Zarook Shareefdeen

**Affiliations:** Department of Chemical and Biological Engineering, American University of Sharjah, Sharjah P.O. Box 26666, United Arab Emirates; zshareefdeen@aus.edu

**Keywords:** biofilter, transient, parameter, VOCs, benzene, toluene, model, validation

## Abstract

Biofilters are biological air-phase packed-bed reactors used for the removal of industrial air pollutants such as volatile organic compounds (VOCs) and odors. Because of the economic and environmental benefits, biofilter technology is preferred in applications such as wastewater treatment plants, waste recycling facilities, and several chemical industries over conventional treatment methods such as adsorption, absorption, and thermal oxidation processes. In order to predict the performance of biofilters, mathematical models under steady-state and transient conditions are needed. The transient biofilter models for gas-phase bioreactors are highly complex, as they involve several parameters that are not easily determined for industrial applications. In this work, a practical transient biofilter model is developed and an analytical solution for the transient model is obtained. When this model is compared with the published but more complex model, this new transient model produces almost the same level of prediction with equal comparisons of experimental data for VOCs, benzene, and toluene. This simple model has fewer parameters and will be very useful and practical for industrial applications for the analysis of transient biofilter performance.

## 1. Introduction

**Figure 1:**
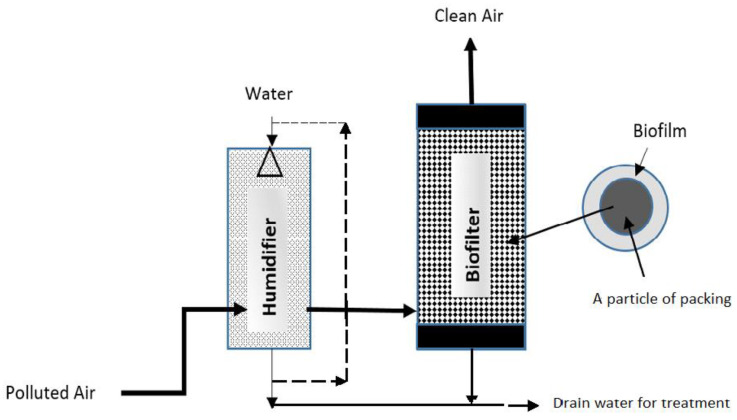
A simplified diagram of the biofilter process.

Biofilters, known as gas-phase bioreactors, are used for the removal of air pollutants, including volatile organic compounds (VOCs) and odor-causing sulfur compounds such as hydrogen sulfide, di-methyl sulfide, ammonia, and amines. In biofilters, incoming polluted air is first humidified and passed through a bed of medium, which can be made using natural (peat, wood chips, saw dust, etc.) or manufactured media particles. On the media particles, the bacteria form biofilms, where pollutants are absorbed and biodegraded. As the air passes through a biofilter along the height, the pollutant concentrations are decreased due to the bio-oxidation of the pollutants. Clean air free of air pollutants exits from the biofilter into the atmosphere in a relatively short empty bed residence time (EBRT) of a few seconds to several minutes. Leachate water (i.e., drain) from the humidifier and the biofilter system is disposed after treatment. The process is depicted in the simplified diagram (Figure 1).

In order to develop a transient biofilter model, mass balances of the air pollutants are made along the biofilter in the gaseous phase as well as mass balances in the biofilms that are formed on the exterior and interior media particles. Ottengraf and Van Den Oever [1] contributed to the initial development of a steady-state biofilter model with a set of assumptions that included the plug flow for the gas phase and the first- or zero-order kinetics of biodegradation in the biofilm, with the assumptions of oxygen availability in biofilms. This model was later extended by Shareefdeen et al. [2] to include oxygen limitation effects and inhibitory kinetic expressions under steady-state conditions. When the inhibitory kinetic effects were considered, the model became more complex with several additional parameters. The extended steady-state model was validated using experimental data on the methanol biofiltration process, in which a peat-perlite mixture was used as the packing medium.

Shareefdeen and Baltzis [3] later presented a detailed transient biofilter model and validated the model with the toluene biofilter performance data. The main improvement was going from steady-state conditions to transient conditions, but still with a single compound, namely toluene. Shareefdeen et al. [4,5] extended their transient biofilter models for a mixture of volatile organic compounds (VOCs, i.e., benzene and toluene) and presented several dynamic studies on the behavior of biofilters under transient conditions. Their work included kinetic interaction effects as well as oxygen limitation effects. Thus, their transient biofilter model is more realistic but has many model parameters that hinder the application of the model to practical conditions.

Baltzis et al. [6] experimentally validated a steady-state model for various VOCs, including benzene, toluene, ethanol, and butanol. Shareefdeen and Shaikh [7] presented a comparative study on the steady-state biofilter models. Shareefdeen et al. [8] later presented a modeling and experimental study on the axial dispersion effects of biofiltration process. Elmrini et al. [9] studied the biofiltration of xylene emissions—bioreactor response to variations in the inlet concentration and gas flow rate. Malakar et al. [10] investigated the toluene removal performance of a biofilter under transient conditions and reported that the Ottengraf mathematical model [1] can predict the theoretical elimination capacities under different regimes of biofilter operations. Salih et al. [11] recently used a novel approach to solve a steady-state biofilter model analytically and they applied the Mann–Green embedded method (MGEM) to obtain an analytical solution for steady-state biofilter models. Rajamanickam et al. [12] recently studied the biofilter performance of benzene under both continuous and transient operations. However, their work was more on experiments rather than modeling. Jawad et al. [13] recently studied the methane removal performance of a biofilter with supporting filter media consisting of composted sawdust. They developed a transient model to predict the methane concentrations at various heights and times. The model equations were solved using a finite difference backward implicit numerical scheme using FORTRAN 77. There are a plenty of studies available on biofilter applications [14,15,16]; however, the studies on practical transient models that can be used by industry are still limited.

The objective of this research work is to develop a transient biofilter model that is simple, analytical, and less complicated with fewer parameters as compared to other published studies. The newer biofilter transient model is expected be more practical for industrial applications and simple to use in determining biofilter performance data under transient conditions. Furthermore, the developed model will have an analytical solution and will be validated with the experimental data obtained from the literature [6] using two compounds, benzene and toluene.

## 2. Theory and Model Development

### 2.1. Model Development

In order to develop a transient biofilter model, the gas phase is assumed to be in plug flow mode and there are no dispersion effects. This assumption eliminates the determination of dispersion coefficients. Taking a control volume along the height (h) of a biofilter and making a mass balance of the air pollutant (i.e., VOC) in the gas phase yields the following equation:

(1) $$\varepsilon \frac{dC_{g}}{dt}=-u_{g}\frac{dC_{g}}{dh}+D_{e}A_{s}{\left[\frac{dC_{l}}{dx}\right]}_{x=0}$$

**Figure 2:**
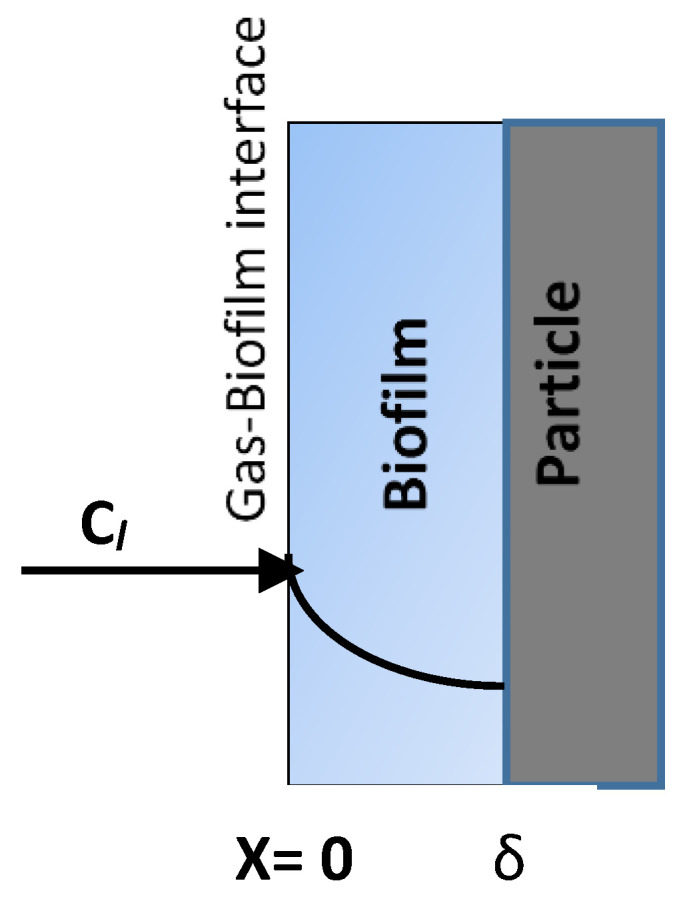
The diagram shows the diffusion of a pollutant into the biofilm at the gas–biofilm interface.

This equation represents the accumulation of the air pollutant within the control volume of the biofilter equal to the net convective mass transfer within the control volume and the amount of pollutant transferred to the biofilm at the gas–biofilm (i.e., liquid phase) interface, which is shown in Figure 2.

The symbols used in the above equation are as follows: *ε* = the void fraction within the biofilter (-), which can be calculated based on the void volume versus the total volume of the biofilter packing media; *C_g_* = the concentration of the pollutant in the gas phase (g·m^−3^); *t* = time (s); *u_g_* = the superficial velocity through the biofilter (m·s^−1^), which can be also calculated as the biofilter media volume (*V*) divided by the cross-section area (*A*) of the biofilter; *h* = the height unit of the biofilter (m); *D_e_* = the effective diffusivity of the pollutant in the biofilm (m·s^−2^), and this parameter (*D_e_*) is related to the diffusivity in the water (*D_w_*) and the biofilm density *X* (Kg·m^−3^). The empirical relationship that relates the diffusivity of the pollutant in the biofilm to the diffusivity in the water is given by [2]:

(2) $$\frac{D_{e}}{D_{w}}=1-\frac{0.43X^{0.92}}{11.19+0.27X^{0.99}}$$

where *A_s_* = the total surface area of the biofilm divided by the biofilter media volume (m^2^·m^−3^); *C_l_* = the concentration of the pollutant in the biofilm (g·m^−3^); *x* = the distance unit in the biofilm (m).

In order to obtain a solution for Equation (1), first we need to determine the concentration gradient ${\left[\frac{dC_{l}}{dx}\right]}_{x=0}$ at the gas–biofilm interface (refer to Figure 2). Based on the previous study [1], the concentration profile (i.e., concentration of a pollutant within the biofilm) for the first-order biodegradation kinetics is given by Equation (3), which can be easily derived. Here, we assume that the pollutant degrades according to the first-order kinetics of biodegradation. However, the method presented in this work can be extended to other kinetic expressions as well.

(3) $$C_{l}=\frac{C_{g}}{H^{\prime }\left(1+e^{-2\delta Ø}\right)}\;\left[e^{Ø\left(x-2\delta \right)}+e^{-Øx}\right]$$

In the above equation, *H*′ = the dimensionless Henry’s constant, which can be easily obtained from the literature and describes the distribution of the pollutant between the gas and biofilm phases (*C_g_*/*C_l_*); *ẟ* = the biofilm thickness (m); Ø is related to the kinetic constant as follows:

(4) $$Ø=\sqrt{{\frac{k_{1}}{D_{e}}}_{}}$$

where *k*_1_ is the first-order biodegradation kinetic constant, which is given by:

(5) $$k_{1}=\frac{{µ}_{mX}}{YK}$$

The evaluation of the first-order kinetic constant k_1_ requires the Monod kinetic parameter constants *K* (g·m^−3^), maximum specific growth rate ${µ}_{m}$(s^−1^), and yield coefficient *Y* (-), which is the ratio of the rate of biomass growth to the consumption of pollutants in the biofilm.

The derivative of the biofilm concentration given by Equation (3) at *x* = 0 (i.e., biofilm interface) yields the following equation:

(6) $${\left[\frac{dC_{l}}{dx}\right]}_{x=0}=-Ø\frac{C_{g}}{H^{\prime }}\tanh\left(Ø\delta \right)$$

The substitution of (6) in Equation (1) yields:

(7) $$\varepsilon \frac{dC_{g}}{dt}=-u_{g}\frac{dC_{g}}{dh}+D_{e}A_{s}-Ø\frac{C_{g}}{H^{\prime }}\tanh\left(Ø\delta \right)$$

For each segment of the biofilter length, ∆*h* (where ∆*h* = *H*/*n*, where *n* is the number of divisions of the biofilter height *H*), Equation (7) can be solved analytically to yield the following equations:

For any segment:

(8) $$C_{gi}\;=C_{g\left(i-1\right)}\gamma_{1}^{}\;[1-\exp\;(-\gamma_{2}\;t){]}^{}$$

To calculate the exit concentration (*C_ge_*) for a given inlet concentration (*C_g_*_0_), the derivation yields the following:

(9) $$C_{ge}\;=C_{g0}\gamma_{1}^{n}\;[1\;-\;\exp\;(-\gamma_{2}\;t){]}^{n}$$

It is important to note that as compared to the other transient biofilter models, this model has an analytical solution with only two model parameters, namely $\gamma_{1}$ and $\gamma_{2}$, which are defined as follows:

(10) $$\gamma_{1}\;={\frac{H^{\prime }u_{g}}{H^{\prime }u_{g}+\left[D_{e}A_{s}\varphi \tan h\left(\delta \varphi \right)\right]∆h\;}}_{\;}$$

(11) $$\gamma_{2}\;={\frac{H^{\prime }u_{g}+[D_{e}A_{s}\varphi \tan h\left(\delta \varphi \right)]∆h\;}{H^{\prime }\varepsilon ∆h}}_{\;}\;\mathrm{or}\;\mathrm{simply}\;\gamma_{2}\;={\frac{u_{g}\;}{\gamma_{1}\varepsilon ∆h}}_{\;}$$

Equations (8)–(11) constitute a simple and practical analytical transient biofilter model that can be solved easily using any open-source programming language, such as Python.

### 2.2. Model Parameters

In order to estimate the model parameters $\gamma_{1}$ and $\gamma_{2},$ we need values for *H*’, *u_g_*, *D_e_*, *A_S_*, δ, ε, and ∆h. In addition to these parameters, we also need to obtain the kinetic parameters *Y*, µ_m_, *K,* and *X* in order to estimate the parameter Ø, which is defined by Equation (4). Most of these parameters are available from [6] and are listed in Table 1.

### 2.3. Comments on the Model Parameters

The parameters used in Table 1 (A) are compound-specific. Thus, one needs to find *H*′, µ_m_, *K*, *Y,* and *D_w_* from the literature for the specific compound under study. It may be a lot easier to get the accurate values of *H*′ and *D_w_* from the literature than the kinetic parameters µ_m_, K, and Y, which are generally obtained from batch kinetic experiments in the lab using a mixed bacterial culture. The bacteria that dominate in a given biofilter may differ from the properties of a mixed bacterial culture and the experimental conditions used in the lab experiments. Therefore, the kinetic parameter values may not completely represent the kinetics of biodegradation in the biofilter. Thus, additional experiments in the biofilter (i.e., lab-scale column or pilot scale) may be required to refine these parameters.

The parameters given in Table 1 (B) are biofilm-specific. The parameters *X_v_*, *A_s_*, and *ẟ* were obtained from the literature [6] for our study and model validations. Although the biofilm density (*X_v_*) is taken as a fixed value of 100 Kg·m^−3^ for model validation, this parameter can have different values from one biofilter to the other, depending on the type of compound that is treated, the biofilter media that is used, the bacteria within the given biofilter, and other factors. The value of *X_v_* also affects the diffusivity (*D_e_*) in the biofilm as per Equation (2) described before. Similarly, the biofilm surface area per unit volume of the biofilter (*A_s_*) and biofilm thickness (*ẟ*) are inter-dependent and vary from one biofilter to the other. Thus, the direct application of these model parameter values in the model prediction of biofilter performance does not always yield accurate results; thus, calibration for these parameters is often needed using lab-scale or pilot-scale biofilter experiments.

The parameters given in Table 1 (C) are specific to the biofilter and biofilter operations. The parameters *C_g_*_0_, *H*, ∆*h*, and *u_g_* can be easily varied to study the effects of the inlet concentration, superficial velocity (*u_g_*), and empty bed residence time (EBRT = *H*/*u_g_*) on the removal performance of the biofilter. Step and random variations in the inlet concentrations or flow rates can be easily adjusted and their responses to the outlet concentrations or removal efficiencies can be easily predicted while maintaining all other parameters at constant values.

## 3. Results and Discussion

**Figure 3:**
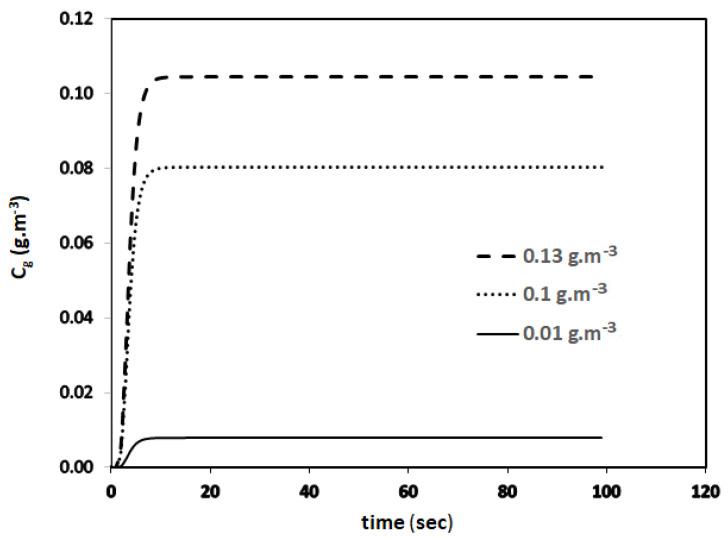
Transient concentration profiles predicated by the new model.

The transient biofilter model represented by Equations (8)–(11) is solved using python programming as well as programming in excel with the parameter values given in Table 1. Python is an open-source programming language that was made to be easy to read and understand. It is also a good choice for a large number of programming problems. Python has become one of the most famous programming languages [17]. The author may be contacted to obtain the source code of the Python program that predicts the data reported in Table 2. The parameters for toluene can be found in [6]. This new transient biofilter model given by Equation (9) is used to predict the concentration profiles given in Figure 3 for three different inlet concentrations of benzene (*C_g_*_0_), 0.13, 0.1, and 0.01 g.m^−3^. For this run, the EBRT value was set at 60 s.

**Figure 4:**
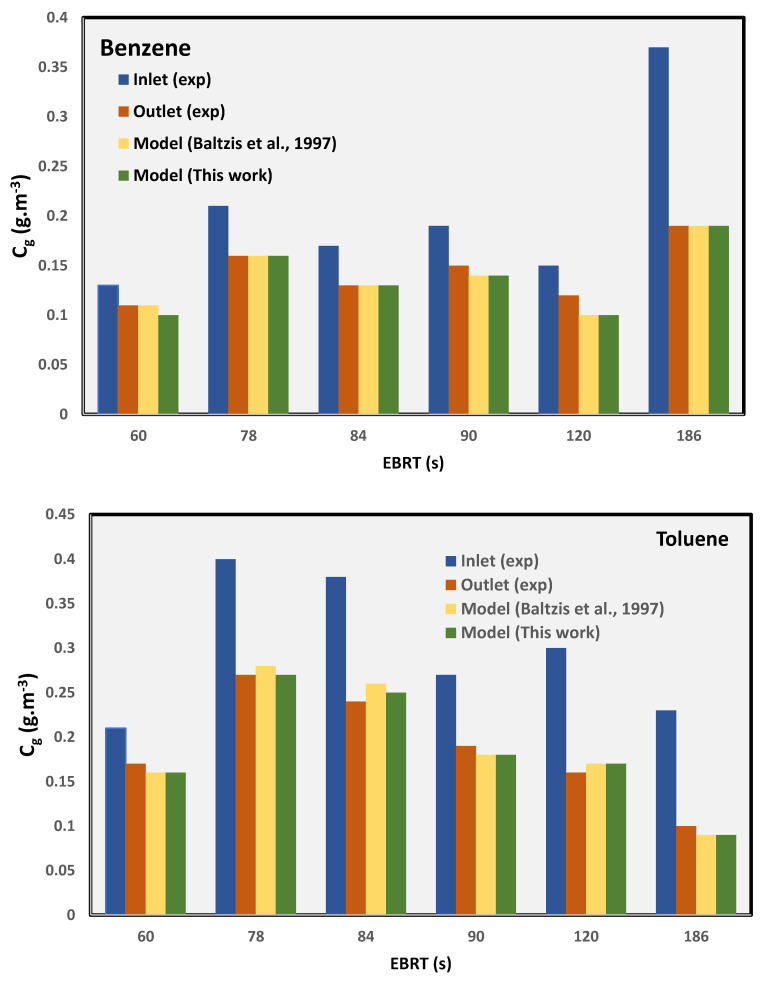
Comparison of model-predicted data against the experimental values [6].

From the transient profiles, the steady-state values (i.e., time → large) of the outlet concentrations can be obtained. The concentration predicted using this new model is compared with the experimental data from the literature at steady-state conditions as well with the model predictions of Baltzis et al. [6] for benzene and toluene. The comparative data are summarized in Table 2 and Figure 4. The comparison of the errors between the two models and the steady-state experimental data shows that the new simplified model, which has fewer parameters, can predict the data reasonably well as compared to Baltzis et al.’s [6] model, which is more complex and has many parameters. Figure 4 shows that the outlet concentrations predicted by the two models are almost identical.

**Figure 5:**
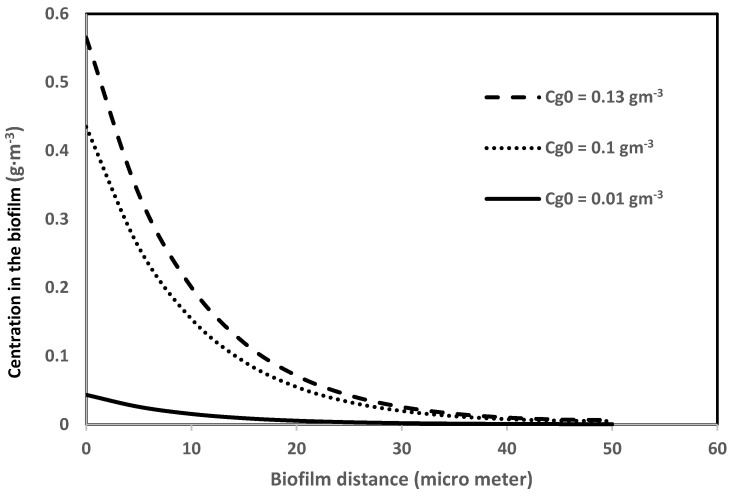
Concentration profiles of benzene in the biofilm in a steady state.

Equation (3) is used to generate biofilm concentration profiles. Figure 5 shows the concentration profiles in the biofilm under the biofilter entrance conditions (i.e., *h* = 0) for the same three different biofilter inlet concentrations of benzene (0.13, 0.1, and 0.01 g·m^−3^). This figure shows that the concentration gradient or mass flux into the biofilm ${\left[\frac{dC_{l}}{dx}\right]}_{x=0}$ decreases as the concentration of the pollutant decreases. The concentration fluxes into the biofilm determine the outlet concentration of the biofilter performance; thus, the accurate determination of this flux is important at every stage (∆*h*) of the biofilter.

**Figure 6:**
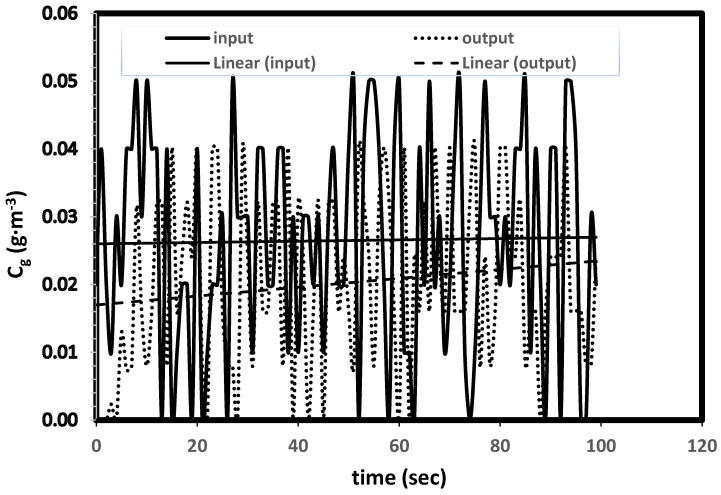
Prediction of biofilter performance with random variations in inlet concentrations.

This transient biofilter model can be easily applied to different transient conditions such as sudden or cycling changes in the inlet concentrations and flowrates. In practical situations, random changes in inlet concentrations are more common than the steady-level of concentrations due to operational, maintenance and schedule changes. In order to demonstrate the model predictive capability, random changes to the inlet concentration levels are introduced. Figure 6 shows the model predicted outlet concentrations. This new model can be easily applied to different transient conditions such as variations in flow rates and step changes in concentrations and can predict responses to the outlet concentration levels and the biofilter performance.

## 4. Conclusions

In this work, a simple transient biofilter model with fewer parameters was developed and validated with experimental data and an analytical solution was derived. The model was solved using both Python programming as well as Excel, which yielded the same results. It is worthwhile mentioning that Baltzis et al. [6] employed the Runge–Kutta algorithm along with the orthogonal collocation technique to numerically solve their biofilter model on the UNIX platform. The parameters used in solving the model were tabulated so that the users can easily verify the results of this study. The performance predictions were compared with the experimental and model-predicted data from a more complex model [6] which has multiple parameters. This simple transient model predicts the biofilter performance at almost the same level of experimental validation using the biofiltration data for two volatile organic compounds (VOC), namely benzene and toluene. The model developed in this study has fewer parameters and is simple to use; thus, engineers and industrial practitioners will find this model more robust for the prediction of transient biofilter performance under various conditions, including for random variations in the inlet concentrations, flow rates, and start-up and shut-down conditions of a biofilter. The transient model developed in this study can be easily extended for the simultaneous removal of multiple pollutants with oxygen limitations and kinetic interaction effects. 

## Figures and Tables

**Table 1:** Parameter values [6] used in the model solutions.

Parameter	Description	Values	Units	Remarks
(A) The parameters in this section are compound-dependent				
*H*′	Dimensionless Henry’s constant	0.23	--	Specific to benzene
µ_m_	Specific growth rate (in Monod expression)	0.68	h^−1^	“
*K*	Kinetic constant (in Monod expression)	12.22	g.m^−3^	“
*Y*	Yield coefficient	0.708	--	“
*D_w_*	Diffusivity of benzene in water	1.04 × 10^−9^	m^2^.s^−1^	“
*D_e_*	Diffusivity of benzene in biofilm	*	m^2^.s^−1^	* Calculated using the values *D_w_*, *X_v_*, and Equation (2).
(B) The parameters in this section are related to the biofilm and biofilter				
*X_v_*	Biofilm density	100	Kg.m^−3^	Specific to biofilm
*A_s_*	Biofilm surface area	40	m^−1^	“
*ẟ*	Biofilm thickness	5.0 × 10^−5^	m	“
(C) The parameters in this section are related to the biofilter operation				
*C_g_*_0_	Inlet concentration to the biofilter	0.13	g.m^−3^	Specific to biofilter operation
H	Height of the biofilter	1.5	m	
∆*h*	A section of a biofilter (if n = 10)	0.15	m	
*u_g_*	Superficial velocity	0.025	m.s^−1^	

**Table 2:** Model validation and comparison with the experimental data from Baltzis et al. [6].

EBRT (s)	Inlet C_go_ (g·m^−3^)	Outlet C_ge_ (g·m^−3^) Experimental	Outlet C_ge_ (g·m^−3^) Baltzis et al. [6]	Error (%) Compared to Exp.	Outlet C_ge_ (g·m^−3^) This Work	Error (%) Compared to Exp.
Benzene						
60	0.13	0.11	0.11	0	0.1	−9.1
78	0.21	0.16	0.16	0	0.16	0
84	0.17	0.13	0.13	0	0.13	0
90	0.19	0.15	0.14	−6.2	0.14	−6.2
120	0.15	0.12	0.1	−16.7	0.1	−16.7
186	0.37	0.19	0.19	0	0.19	0
Toluene						
60	0.21	0.17	0.16	−5.9	0.16	−5.9
78	0.4	0.27	0.28	3.7	0.27	0
84	0.38	0.24	0.26	8.3	0.25	4.2
90	0.27	0.19	0.18	−5.3	0.18	−5.3
120	0.3	0.16	0.17	6.2	0.17	6.2
186	0.23	0.1	0.09	−10	0.09	−10
